# Comprehensive assessment of supratentorial and infratentorial volumes in infants with myelomeningocele with and without Chiari malformation type II

**DOI:** 10.1007/s00234-024-03514-9

**Published:** 2024-12-04

**Authors:** Hiroaki Hashimoto, Makoto Shimada, Osamu Takemoto, Yasuyoshi Chiba

**Affiliations:** 1 Department of Neurosurgery, Osaka Women’s and Children’s Hospital, 840 Murodocho, Izumi, Osaka 594–1101 Japan; 2 Department of Neurological Diagnosis and Restoration, Graduate School of Medicine, Osaka University, Suita, Osaka Japan; 3 Department of Radiology, Osaka Women’s and Children’s Hospital, Izumi, Osaka Japan

**Keywords:** Chiari malformation type II, CT, Myelomeningocele, Postnatal repair, Volumetric measurement

## Abstract

**Purpose:**

Chiari malformation type II (CM-II) is a congenital anomaly commonly associated with myelomeningocele (MMC), a severe form of open spina dysraphism. This study aimed to evaluate both supratentorial and infratentorial volumes in MMC infants with and without CM-II.

**Methods:**

We conducted a single-center, retrospective study of 52 MMC infants treated between April 2006 and July 2023. Infants were classified as non-CM-II or CM-II based on the presence of cerebellar displacement. All patients underwent computed tomography (CT) at 0 months of age. Volumetric parameters included intracranial volume (ICV), lateral ventricles volume (LVV), posterior cranial fossa volume (PCFV), cerebellum volume (CBMV), and brainstem volume (BSV). LVV represented supratentorial structures, while PCFV, CBMV, and BSV represented infratentorial structures.

**Results:**

CM-II was diagnosed in 30 infants (57.7%). Correlation analysis revealed significant negative correlations between supratentorial (LVV) and infratentorial volumes (PCFV, CBMV, and BSV), and positive correlations among volumes within the same space(e.g., PCFV, CBMV, and BSV). CM-II infants exhibited significantly larger ICV (*p* = 0.04) and LVV (*p* < 0.001), but smaller PCFV (*p* < 0.001) and CBMV (*p* < 0.001) than non-CM-II infants. LVV was the best predictor for distinguishing non-CM-II from CM-II (area under the curve = 0.91).

**Conclusion:**

This study identified positive correlations within the same space and negative correlations between supratentorial and infratentorial volumes. LVV emerged as a critical indicator of CM-II, reflecting the relationship between reduced infratentorial space and enlarged supratentorial ventricles (hydrocephalus). These findings provide insights into the pathophysiology and clinical implications of CM-II in MMC patients.

**Supplementary Information:**

The online version contains supplementary material available at 10.1007/s00234-024-03514-9.

## Introduction

Myelomeningocele (MMC) is the most severe form of open spina dysraphism [1], accounting for 98.8% of open spina dysraphism cases [2]. Open spina dysraphism is characterized by the exposure of neural tissue and/or meninges to the external environment through a congenital bony defect [2]. Chiari malformation type II (CM-II) is a congenital anomaly closely associated with MMC [3, 4], characterized by the downward displacement of the cerebellar vermis, tonsils, and medulla through the foramen magnum into the upper cervical spinal canal [5, 6]. For the objective assessment of CM-II using magnetic resonance imaging (MRI), previous studies have proposed grading systems that evaluate the visibility of the fourth ventricle or cisterna magna and the presence or absence of cerebellar displacement below the foramen magnum [7–9].

Previous studies have reported CM-II in nearly all MMC cases [5, 10, 11], with symptomatic CM-II occurring in 10–30% of these patients [12, 13]. The most common symptom is swallowing dysfunction [6]. Infants and children under two years of age often present with cranial nerve and brainstem signs, with respiratory difficulties being potentially fatal [14]. The rate of CM-II decompression surgeries ranges from 9 to 19% [12, 15].

In fetuses with MMC, cerebrospinal fluid (CSF) leakage into the sac or amniotic fluid is thought to lead to hindbrain herniation, known as CM-II [16]. This herniation is thought to induce hydrocephalus through increased resistance to cerebral venous outflow or abnormalities in CSF absorption or flow [17–20]. Therefore, it can be stated that a small posterior cranial fossa, accompanied by hindbrain herniation, is a consequence of MMC, while hydrocephalus is a consequence of the small posterior cranial fossa. In MMC cases, a small posterior cranial fossa and hydrocephalus are observed in 84% [21] and 85–90% of cases, respectively [5]. Hydrocephalus is frequently associated with symptomatic CM-II [22]. These results suggest a close relationship between infratentorial structural malformations (such as a small posterior cranial fossa) and supratentorial structural malformations (such as enlarged supratentorial ventricles).

Despite suggesting a close relationship between infratentorial and supratentorial malformations, previous studies have focused only on the area (mm^2^) [23] or volume (mm^3^) [24, 25] of the posterior cranial fossa in CM-II patients. They did not provide quantitative data on supratentorial structures. This study aims to provide a comprehensive volumetric assessment of both supratentorial and infratentorial structures in MMC infants. Additionally, we classified MMC infants with cerebellar displacement as CM-II and those without as non-CM-II, aiming to demonstrate volumetric differences between the two groups. We analyzed head computed tomography (CT) images from MMC infants who underwent postnatal repair surgery. To minimize the growth effects, we used only CT images obtained at 0 months of age.

## Materials and methods

### Patients and study setting

This retrospective study included patients with MMC treated at our department between April 2006 and July 2023. All patients underwent head and spinal CT imaging after birth and received postnatal repair surgery at our department. The first head CT, obtained at 0 months of age, was used for intracranial volumetric evaluation. CT scans were performed using Canon Aquilion until 2011, and Canon Aquilion One (Canon Medical Systems, Otawara, Japan) from 2012 onwards. Five different CT protocols were employed, as detailed in Supplemental Table 1.

**Fig. 1:**
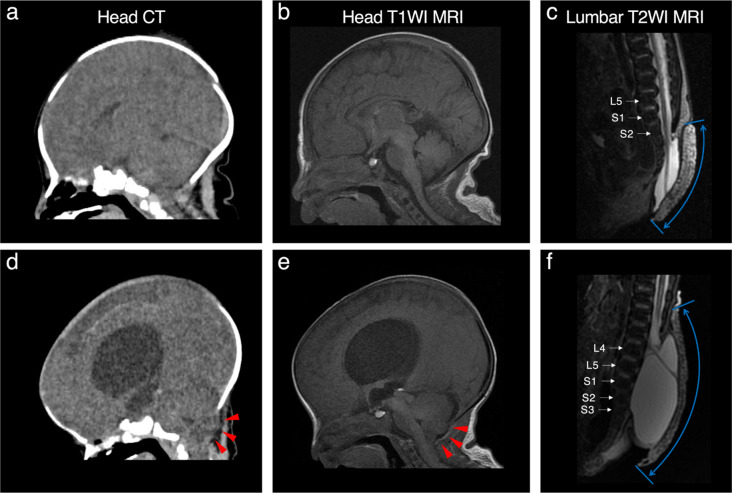
Representative CT and MRI images from non-CM-II or CM-II infants. For a non-CM-II infant, sagittal midline views of head CT (obtained on day 0 after birth) (**a**), head T1WI MRI (day 4) (**b**), and lumbar T2WI MRI (day 0) (**c**) are shown. No cerebellar displacement is observed. The MMC lesion extends from S3 to S5, classified as a sacral type. For a CM-II infant, sagittal midline views of CT (day 0) (**d**), head T1WI MRI (day 7) (**e**), and lumbar T2WI MRI (day 0) (**f**) are presented. Cerebellar displacement through the foramen magnum is observed on both head CT and MRI (indicated by red arrowheads). The MMC lesion extends from L4 to S3, classified as a lower lumbar type. The areas indicated by blue arrows in the lumbar T2WI MRI images (**c** and **f**) show gauzes protecting the MMC lesions. CM-II, Chiari malformation type II; CT, computed tomography; MMC, myelomeningocele; MRI, magnetic resonance imaging; T1WI, T1-weighted image; T2WI, T2-weighted image

Based on previous studies [7–9], we defined four grades as follows: grade 0, normal; grade 1, visible fourth ventricle and cisterna magna without cerebellar displacement below the foramen magnum, with the possible vertical orientation of the tentorium and the presence of tectal beaking; grade 2, cerebellar displacement with effacement of the fourth ventricle but a patent cisterna magna; and grade 3, cerebellar displacement with effacement of both the cisterna magna and the fourth ventricle. In this study, grades 0 and 1 were classified as non-CM-II, while grades 2 and 3 were classified as CM-II, primarily based on the presence of cerebellar displacement (Fig. 1).

Radiologists at our hospital determined the presence of cerebellar displacement in MMC infants based on the first head CT. If cerebellar displacement was unclear on the CT, a head MRI obtained at 0 months was used to support the diagnosis.

### Data collection

We analyzed medical variables, including sex, gestational week, birth weight, fetal diagnosis, cesarean section, days until CT imaging, days until MMC repair, and the necessity for a ventriculoperitoneal shunt (VPS). The necessity for a VPS was determined by neurosurgeons at our department based on a tense fontanelle or increasing head circumference [26]. MMC lesions were categorized into four types based on the involved vertebrae: thoracic (involving at least one thoracic vertebra), upper lumbar (1st and 3rd lumbar vertebrae), lower lumbar (mainly among 3rd and 5th lumbar vertebrae), and sacral (localized to sacral vertebra). A lumbosacral type, where the lesion ranges from the lower lumbar to sacral vertebrae (e.g., from the fifth lumbar to the third sacral vertebrae), was categorized as lower lumbar (Fig. 1). A lumbar MRI or CT performed before closure surgery was used to assess the number of vertebrae affected by MMC lesions and the presence of lumbar syringomyelia. The surface longitudinal length of the MMC sacs was measured using a lumbar MRI at 0 months. MMC lesions were defined as neural placode or impairment of epithelialization.

### Volumetric evaluation

**Fig. 2:**
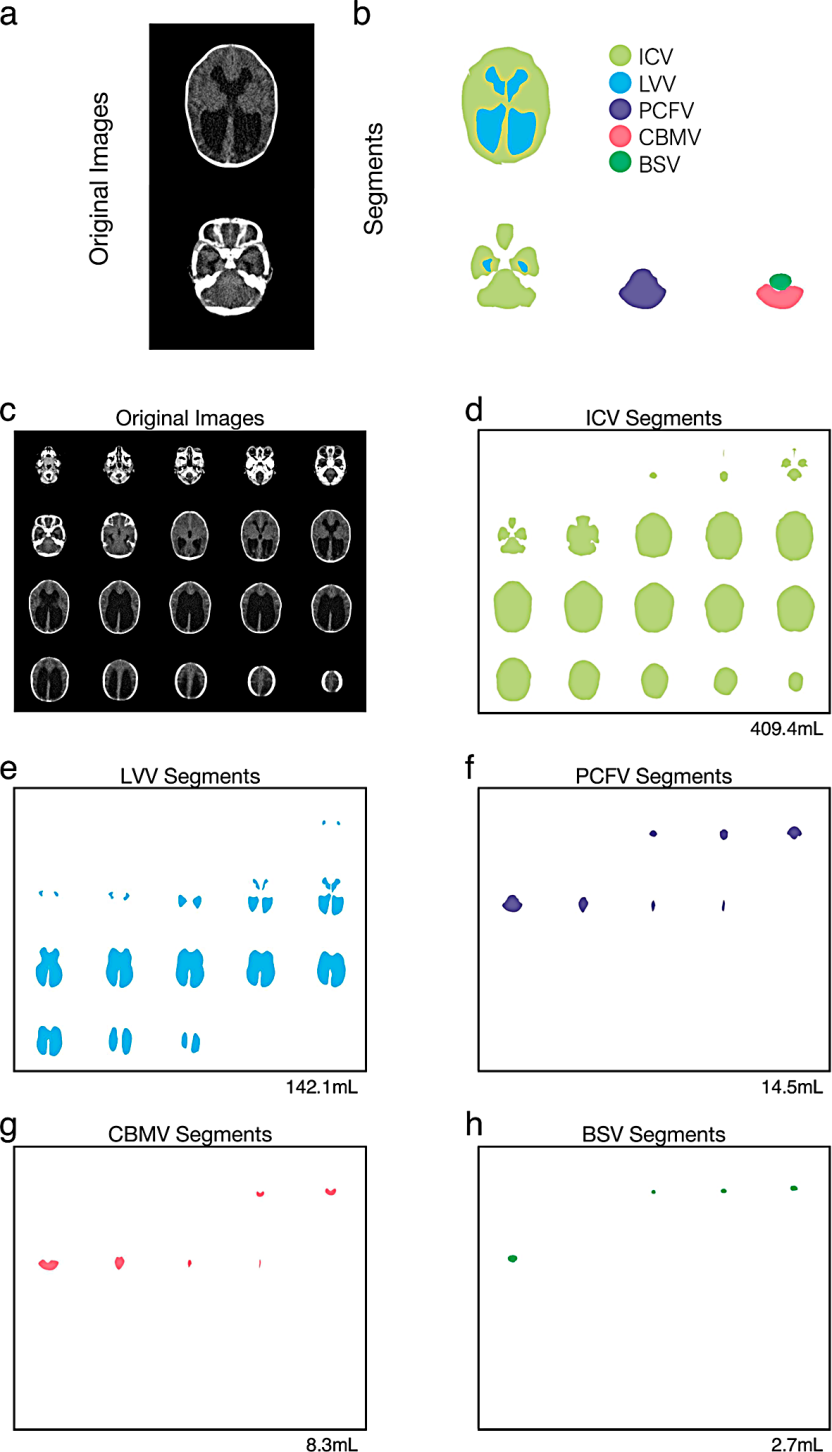
Representative segmentations. Original head CT images and segmentations of intracranial volume (ICV), lateral ventricles volume (LVV), posterior cranial fossa volume (PCFV), cerebellum volume (CBMV), and brainstem volume (BSV) are shown. Representative CT images and their corresponding segmentations are presented (**a** and **b**). All CT images and segmentations are displayed (**c**, **d**, **e**, **f**, **g**, and **h**). The CT images and segmentations are from a CM-II infant, the same infant presented in Fig. 1d and e, and 1f. The calculated volumes for each segmentation are presented in the bottom right corner of each panel. CM-II, Chiari malformation type II; CT, computed tomography

We defined five parameters for the quantitative assessment of intracranial structures: intracranial volume (ICV), lateral ventricles volume (LVV), posterior cranial fossa volume (PCFV), cerebellum volume (CBMV), and brainstem volume (BSV). LVV represented the volume of the bilateral ventricles, and BSV included the medulla oblongata and the pons. Head CT data in Digital Imaging and Communication in Medicine (DICOM) format were imported into MATLAB R2023a (MathWorks, Natick, MA, USA). The target areas were manually segmented using the image segmenter app in MATLAB. Representative segmentation of ICV, LVV, PCFV, CBMV, and BSV are shown in Fig. 2. We counted the total number of pixels within segmentations, and to obtain volume values (in milliliter, mL), the total number of pixels was multiplied by the volume of one pixel. This method aligns with our previous studies [27–31].

Since we used head CT scans obtained at 0 months, it was difficult to differentiate between the cervical spine cord and herniate cerebellum (Supplemental Fig. 1). Therefore, any brain tissue herniating into the spinal canal through the foramen magnum was excluded from segmentation. It is important to note that this study measured structural volumes only within enclosed spaces, such as the intracranial cavity.

### Statistical analyses

Categorical data were presented as frequencies (percentages), and continuous variables were expressed as mean ± standard deviation for normally distributed variables and as median with 1st-3rd quartiles for non-normally distributed variables. Spearman correlation coefficients were used to assess parameter correlations, and the chi-squared test was used to compare categorical variables. The unpaired T-test was employed for parametric distributions, and the Wilcoxon rank-sum test was utilized for non-parametric distributions. Bonferroni correction was applied for multiple comparisons. P-values < 0.05 and corrected p-values (cp.) < 0.05 were considered statistically significant. The receiver operating characteristic (ROC) curve was used to classify two groups, determining the area under the curve (AUC) and cut-off values (COV) using the maximal Youden index (sensitivity + specificity– 1). Statistical analyses were performed using MATLAB R2023a’s Statistical and Machine Learning Toolbox.

## Results

### Baseline characteristics

We enrolled 52 Japanese infants with MMC (27 females, 51.9%) (Table 1), with a median follow-up of 8.11 years (3.95–11.66) as of September 30, 2023. The most common type of MMC was sacral (51.9%). The mean surface longitudinal length of the MMC sacs was 41.23 ± 18.66 mm. Closure surgeries were performed one day after birth (median 1.00 days). Lumbar syringomyelia was observed in 38.5% of cases, and 71.2% of infants required VPS. This population is the same as in our previous study [31].

**Table 1:** Baseline data of enrolled infants with MMC and comparison between MMC without CM-II and with CM-II, and between CM-II without operation and with operation

	Total (*n* = 52)		non-CM-II (*n* = 22)	CM-II (*n* = 30)	*p* values		non-Operation with CM-II (*n* = 26)	Operation with CM-II (*n* = 4)	*p* values
**Sex (** ***n*** **)**									
Male (%)	25 (48.1)		11 (50.0)	14 (46.7)	0.81		14 (53.8)	0 (0.0)	*0.04
Female (%)	27 (51.9)		11 (50.0)	16 (53.3)			12 (46.2)	4 (100.0)	
Gestational week *1	38.10 ± 1.50		38.52 ± 1.63	37.80 ± 1.3	0.09		37.96 ± 1.28	36.75 ± 1.50	0.09
**Weight at birth (g)**	2870.46 ± 547.15		2932.18 ± 437.72	2825.20 ± 618.65	0.49		2867.00 ± 578.68	2553.50 ± 891.98	0.35
**Fetal diagnosis (n)**									
Yes (%)	37 (71.2)		11 (50.0)	26 (86.7)	*0.004		23 (88.5)	3 (75.0)	0.46
No (%)	15 (28.8)		11 (50.0)	4 (13.3)			3 (11.5)	1 (25.0)	
Caesarean section*2 (n)									
Yes (%)	24 (50.0)		7 (35.0)	17 (60.7)	0.08		15 (62.5)	2 (50.0)	0.64
No (%)	24 (50.0)		13 (65.0)	11 (39.3)			9 (37.5)	2 (50.0)	
**Days of life at the initial CT**	0.00 (0.00–1.00)		0.50 (0.00–1.00)	0.00 (0.00–1.00)	0.40		0.00 (0.00–1.00)	0.50 (0.00–1.00)	0.75
**Days of life at MMC repair**	1.00 (0.50–2.00)		1.00 (0.00–2.00)	1.00 (1.00–2.00)	0.55		1.00 (1.00–2.00)	1.50 (0.50–2.50)	0.63
**Days from the CT to MMC repair**	1.00 (0.00–1.00)		0.00 (0.00–1.00)	1.00 (0.00–1.00)	0.13		1.00 (0.00–1.00)	1.00 (0.00–2.00)	0.76
**MMC lesion type (n)**									
Thoracic type (%)	12 (23.1)		1 (4.5)	11 (36.7)	*<0.001		9 (34.6)	2 (50.0)	0.50
Upper lumbar type (%)	3 (5.8)		0 (0.0)	3 (10.0)			3 (11.5)	0 (0.0)	
Lower lumbar type (%)	10 (19.2)		1 (4.5)	9 (30.0)			7 (26.9)	2 (50.0)	
Sacral type (%)	27 (51.9)		20 (90.9)	7 (23.3)			7 (26.9)	0 (0.0)	
**MMC lesion vertebrae count**	3.50 (3.00–5.50)		3.00 (2.00–3.00)	5.00 (3.00–8.00)	*<0.001		5.00 (3.00–8.00)	5.00 (3.00–7.00)	0.78
**Syringomyelia (n)**									
Yes (%)	20 (38.5)		7 (31.8)	13 (43.3)	0.40		12 (46.2)	1 (25.0)	0.43
No (%)	32 (61.5)		15 (68.2)	17 (56.7)			14 (53.8)	3 (75.0)	
**Necessity of VPS (n)**									
Yes (%)	37 (71.2)		8 (36.4)	29 (96.7)	*<0.001		25 (96.2)	4 (100.0)	0.69
No (%)	15 (28.8)		14 (63.6)	1 (3.3)			1 (3.8)	0 (0.0)	

Mean and standard deviation values are presented for parametrically distributed variables, analyzed using unpaired T-tests. For non-parametric variables, median values along with the 1st and 3rd quartiles are provided, and analysis is conducted using the Wilcoxon rank-sum test. Categorical variables are compared using the chi-squared test. Statistical significance is determined at *p* < 0.05, denoted by an asterisk

CM-II, Chiari malformation type II; CT, computed tomography; MMC, myelomeningocele; VPS, ventriculoperitoneal shunt

*1 Missing data were observed in one case; the result was obtained from 51 cases

*2 Missing data were observed in four cases; the results were obtained from 48 cases

Infants with MMC were born at an average of 38.10 gestational weeks (with one data point missing, Table 1). Forty-three infants (82.7%) were born at term (37 weeks 0 days through 41 weeks 6 days), while two infants were born at 35 gestational weeks and six infants at 36 gestational weeks. The first CT scans after birth, used for volumetric calculations, were performed on day 0 (median: 0.00 days). Of these, 47 scans (90.4%) were obtained before closure surgery, while five were performed after surgery. For nine cases, both CT and MRI were needed to assess cerebellar displacement (Supplemental Fig. 2, which details the radiologists’ experience in pediatric radiology). A total of 30 cases (57.7%) were classified as CM-II. Four cases (7.7%) required foramen magnum decompression with C1 laminectomy due to respiratory difficulties (Supplemental Fig. 3), with surgery performed at an average of 3.50 ± 3.42 months after birth.

Prenatal MMC diagnosis was made in 37 cases (71.2%); among these, fetal MRIs were performed in 32 cases (86.5%). In 26 cases (81.3%), the prenatal diagnosis of CM-II or non-CM-II was consistent with the postnatal diagnosis (Supplemental Fig. 4).

Significant differences were found when comparing non-CM-II and CM-II cases. In MMC with CM-II, the rate of fetal diagnosis and VPS were higher (chi-square test, *p* = 0.004, and *p* < 0.001, respectively), and the MMC lesion vertebrae count was higher (Wilcoxon rank-sum test, *p* < 0.001). The type of MMC lesions also differed significantly (chi-square test, *p* < 0.001): 90.9% of MMC cases without CM-II were sacral type, compared to only 23.3% of those with CM-II. No significant differences were found between CM-II cases without and with the operation, except for sex (chi-square test, *p* = 0.04) (Table 1). All four operated cases were female, possibly influencing the results.

### Correlation analysis between continuous variables

We analyzed correlations between ICV, LVV, PCFV, CBMV, BSV, and MMC lesion vertebrae count (Table 2). Significant positive correlations were found between ICV and LVV (*r* = 0.67, *p* < 0.001), as well as among the infratentorial structures: PCFV and CBMV (*r* = 0.94, *p* < 0.001), PCFV and BSV (*r* = 0.68, *p* < 0.001), and CBMV and BSV (*r* = 0.67, *p* < 0.001). Significant negative correlations were observed between supratentorial (LVV) and infratentorial structures (PCFV, CBMV, and BSV), including LVV and PCFV (*r* = -0.69, *p* < 0.001), LVV and CBMV (*r* = -0.56, *p* < 0.001), and LVV and BSV (*r* = -0.30, *p* < 0.03). The MMC lesion vertebrae count demonstrated significant positive correlations with ICV (*r* = 0.51, *p* < 0.001) and LVV (*r* = 0.66, *p* < 0.001), along with a significant negative correlation with PCFV (*r* = -0.37, *p* = 0.006).

**Table 2:** Correlation coefficients between parameters

Parameters	ICV	LVV	PCFV	CBMV	BSV	MMC lesion vertebrae count
ICV			-0.14 (*p* = 0.32)	-0.05 (*p* = 0.72)		
LVV	0.67 (* ***p*** ** < 0.001)**		-0.69 (* ***p*** ** < 0.001)**	-0.56 (* ***p*** ** < 0.001)**	-0.30 (* ***p*** ** = 0.03)**	
PCFV						**-0.37** (* ***p*** **= 0.006)**
CBMV			0.94 (* ***p*** ** < 0.001)**			-0.26 (*p* = 0.06)
BSV	0.13 (*p* = 0.35)		0.68 (* *p* ** < 0.001)**	0.67 (* ***p*** ** < 0.001)**		-0.13 (*p* = 0.34)
MMC lesion vertebrae count	0.51 (* ***p*** ** < 0.001)**	0.66 (* ***p*** ** < 0.001)**				

Correlation coefficients between parameters are shown with corresponding p-values. Significant p-values are denoted with an asterisk (*). Positive correlation coefficients are demonstrated below the diagonal line, and negative correlation coefficients are presented above the diagonal line

BSV, brainstem volume; CBMV, cerebellum volume; ICV, intracranial volume; LVV, lateral ventricles volume; MMC, myelomeningocele; PCFV, posterior cranial fossa volume

### Intracranial volumetric assessment

The median values of five intracranial parameters and the ratio of the sum of CBMV and BSV divided by PCFV are presented in Table 3. Results from all infants (*n* = 52), non-CM-II infants (*n* = 22), CM-II infants (*n* = 30), non-operation CM-II infants (*n* = 26), and operation CM-II infants (*n* = 4) are included. Significant differences were observed between non-CM-II and CM-II groups, but not between non-operation and operation groups.

**Table 3:** Baseline volumetric data and comparison between MMC without Chiari malformation and with Chiari malformation, and between Chiari malformation without operation and with operation

	Total		non-CM-II (*n* = 22)	CM-II (*n* = 30)	*p* values		non-Operation with CM-II (*n* = 26)	Operation with CM-II (*n* = 4)	*p* values
ICV (mL)	407.50 (353.55–490.45)		376.05 (344.00–421.20)	431.20 (368.80–594.10)	*0.04		431.20(373.90–594.10)	400.50(314.50–1049.90)	0.48
LVV (mL)	33.18 (6.32–120.67)		4.67 (3.16–19.75)	79.57 (31.40–219.56)	*<0.001		79.57 (41.56–219.56)	75.11 (30.58–723.45)	0.98
PCFV (mL)	21.35 (16.65–25.60)		25.60 (21.80–27.90)	17.35 (14.50–21.40)	*<0.001		18.45 (14.70–21.40)	12.80 (8.30–19.15)	0.17
CBMV (mL)	13.79 (10.62–16.00)		15.75 (13.75–18.51)	11.72 (8.61–14.80)	*<0.001		12.28 (9.99–14.80)	7.09 (4.98–11.99)	0.12
BSV (mL)	2.86 (2.49–3.33)		3.04 (2.64–3.26)	2.58 (2.19–3.40)	0.05		2.64 (2.39–3.40)	1.93 (1.76–2.72)	0.08
(CBMV + BSV) / PCFV	0.79 (0.76–0.83)		0.76 (0.69–0.82)	0.80 (0.78–0.84)	*0.01		0.80 (0.78–0.84)	0.79 (0.71–0.87)	0.69

Median values with 1st-3rd quartiles are presented for each segmentation data. Statistical significance is determined using the Wilcoxon rank-sum test at *p* < 0.05, denoted by an asterisk

BSV, brainstem volume; CBMV, cerebellum volume; CM-II, Chiari malformation type II; ICV, intracranial volume; LVV, lateral ventricles volume; MMC, myelomeningocele; PCFV, posterior cranial fossa volume

The CM-II group had significantly higher values for ICV (*p* = 0.04), LVV (*p* < 0.001), and the ratio of CBMV + BSV to PCFV (*p* = 0.01), and significantly lower values for PCFV (*p* < 0.001) and CBMV (*p* < 0.001). Thus, the CM-II group exhibited larger intracranial cavity and supratentorial structures, and smaller infratentorial structures than the non-CM-II group. Individual volumetric data in three-dimensional space (LVV, PCFV, and MMC lesion vertebrae count) revealed two distinct groups: non-CM-II and CM-II (Supplemental Fig. 5).

**Fig. 3:**
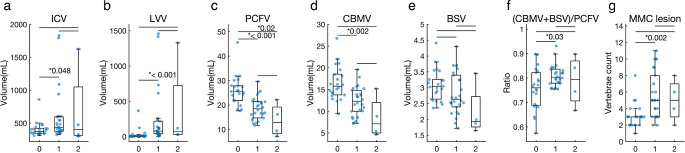
Differences among three MMC groups. The box-and-whisker plots overlaid with beeswarm plots are presented for three groups: (0) non-CM-II group (*n* = 22), (1) CM-II not requiring operation group (*n* = 26), and (2) CM-II requiring operation group (*n* = 4). The numbers correspond to those presented on the x-axis. Results are presented for intracranial volume (ICV) (**a**), lateral ventricles volume (LVV) (**b**), posterior cranial fossa volume (PCFV) (**c**), cerebellum volume (CBMV) (**d**), brainstem volume (BSV) (**e**), the ratio of CBMV + BSV to PCFV (**f**), and MMC lesion vertebrae count (**g**). The Wilcoxon rank-sum test is used, and acquired p-values are corrected by Bonferroni correction, multiplied by three, for multiple comparisons. Statistically significant corrected-p values < 0.05 are denoted with an asterisk. CM-II, Chiari malformation type II; MMC, myelomeningocele

Distribution plots with box-and-whisker plots for non-CM-II (*n* = 22), non-operation in CM-II (*n* = 26), and operation CM-II (*n* = 4) showed that non-CM-II had statistically lower ICV, LVV, the ratio of CBMV + BSV to PCFV, and MMC lesion vertebrae count than non-operation CM-II (c.*p* = 0.048, < 0.001, 0.03, and 0.002 respectively, Wilcoxon rank-sum test corrected by Bonferroni correction, as shown below) (Fig. 3a, b and f, and 3g). Conversely, non-CM-II had higher CBMV (c.*p* = 0.02) (Fig. 3d). Non-CM-II had higher PCFV than both non-operation and operation CM-II groups (c.p = < 0.001 and 0.02, respectively) (Fig. 3c).

### ROC analysis

**Fig. 4:**
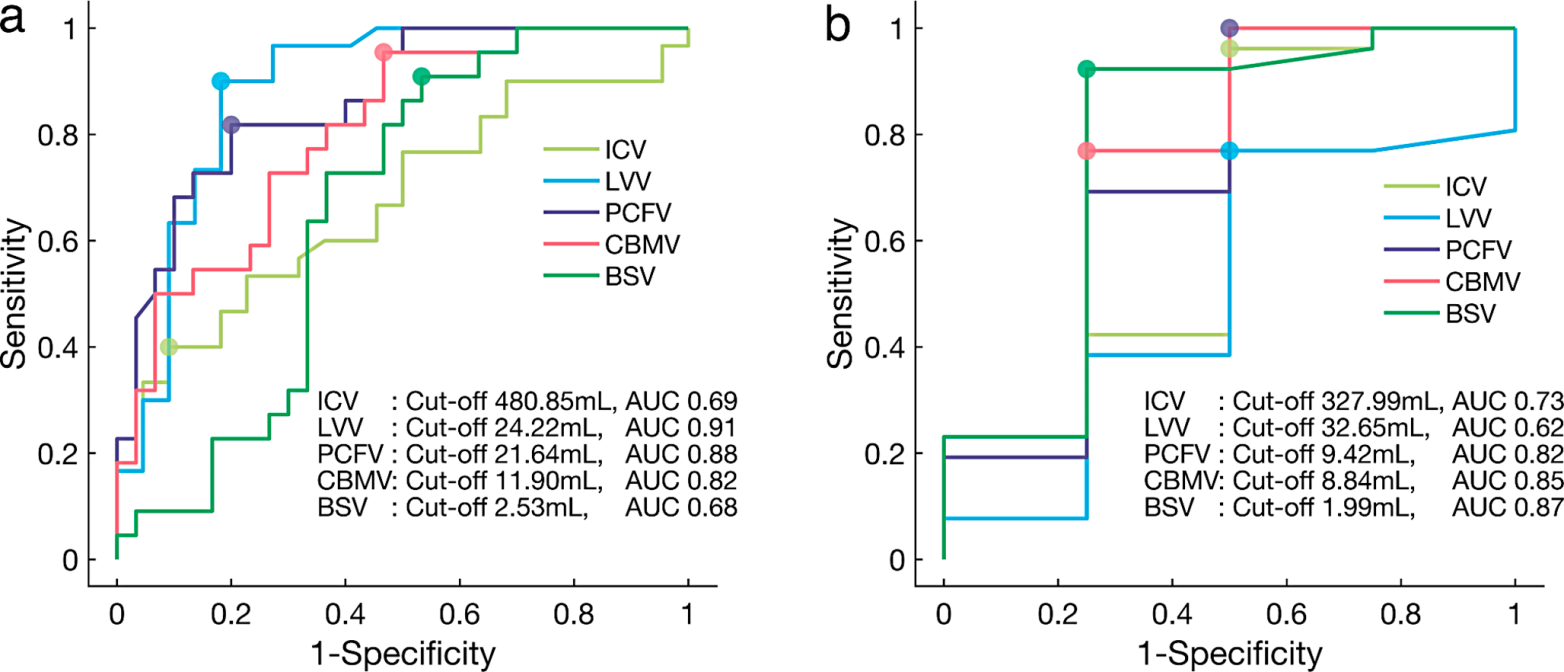
Receiver operating characteristic (ROC) curves. ROC curves were generated using the five volumetric parameters-intracranial volume (ICV), lateral ventricles volume (LVV), posterior cranial fossa volume (PCFV), cerebellum volume (CBMV), and brainstem volume (BSV)-to discriminate between non-CM-II infants (*n* = 22) from CM-II infants (*n* = 30) (**a**), and to distinguish CM-II infants who did not require surgery (*n* = 26) from those who did (*n* = 4) (**b**) CM-II, Chiari malformation type II; MMC, myelomeningocele

ROC analysis distinguished non-CM-II (*n* = 22) and CM-II (*n* = 30) using five volumetric parameters. LVV had the highest AUC (0.91, COV = 24.22mL, sensitivity 0.90, specificity 0.82). PCFV was second (AUC = 0.88, COV = 21.64mL sensitivity 0.82, specificity 0.80) (Fig. 4a).

For differentiating CM-II cases without operation (*n* = 26) and with operation (*n* = 4), LVV had the lowest AUC (0.62, COV = 32.65mL sensitivity 0.77, specificity 0.50). High performance was demonstrated by infratentorial structures: PCFV (AUC = 0.82, COV = 9.42mL, sensitivity 1.00, specificity 0.50), CBMV (AUC = 0.85, COV = 8.84mL, sensitivity 0.77, specificity 0.75), and BSV (AUC = 0.87, COV = 1.99mL, sensitivity 0.92, specificity 0.75) (Fig. 4b), with BSV showing the highest performance.

### DICOM slice width

Due to the retrospective nature of this study, we were unable to maintain consistency in the slice width of CT scans. The DICOM slice width ranged from 1 mm to 6 mm, with a median of 5.00 (5.00–5.33) mm, and 80.8% of the scans had a slice width of 5 mm (Supplemental Table 1). Slice width correlated significantly positively with ICV (*r* = 0.38, *p* = 0.006) and LVV (*r* = 0.40, *p* = 0.003). However, no significant correlation was found between slice width and infratentorial structures like PCFV (*r* = -0.04, *p* = 0.78), CBMV (*r* = 0.05, *p* = 0.72), and BSV (*r* = 0.07, *p* = 0.59).

## Discussion

Previously, we established baseline intracranial volume data for MMC infants in relation to hydrocephalus [31]. Using the same cohort and focusing on CM-II, this study aimed to assess both supratentorial and infratentorial structural volumes comprehensively. Positive correlations were observed among infratentorial structures, while negative correlations were found between supratentorial and infratentorial structures. Additionally, statistical differences were noted in the volumetric comparison between non-CM-II and CM-II infants. Finally, ROC analysis demonstrated LVV as the best marker for distinguishing non-CM-II from CM-II.

Previous volume assessments of the posterior cranial fossa have been conducted in both normal populations [29, 32] and those with conditions like achondroplasia [33] and Chiari malformation type I [34]. However, volumetric assessments for CM-II are limited and have primarily focused on infratentorial structures [24, 25]. This study provides comprehensive volumetric data of both supratentorial and infratentorial structures in relation to CM-II infants. Since an infant’s ICV grows rapidly, nearly doubling by one year of age [30], we used head CT data obtained at 0 months to minimize the effects of growth and ensure consistent measurement conditions. Although our volumetric data were smaller than those previously reported [25], this discrepancy might be due to differences in segmentation methods. Unlike the previous study [25], we excluded the cerebellum herniating into the spinal canal from volume calculation. This exclusion was necessary because we could not differentiate the herniating cerebellum from the cervical spinal cord, which is a notable limitation of this study. Additionally, as 82.7% of infants were born at term, the influence of gestational weeks is expected to be minimal.

The CM-II morbidity rate among MMC infants in this study (57.7%) differs from historical reports, which associate nearly all MMC cases with CM-II [5, 10, 11]. Grading systems have been used to objectively describe the severity of CM-II [7–9], and we referred to these studies. We classified MMC infants into non-CM-II or CM-II based on the presence of cerebellar displacement. Consequently, MMC infants with no cerebellar displacement but with other CM-II-specific findings, such as tectal beaking, were classified as non-CM-II. The reason for using these diagnostic criteria is that, since we only used CT and MRI imaging obtained at 0 months, it was difficult to accurately detect CM-II-specific findings other than cerebellar displacement. Additionally, considering the increasing incidence of hydrocephalus associated with MMC over time [18, 31, 35, 36], CM-II incidence and severity could also vary postnatally. These factors may contribute to the lower CM-II rate observed in our study. Nonetheless, this is a notable limitation.

Correlation analysis confirmed several empirically known relationships. Significant positive correlations were found among volumetric parameters within the same space, such as the infratentorial space: PCFV, CBMV, and BSV. Positive correlations between ICV and LVV have been reported in the pediatric population [30], and our results showed similar positive relationships. This suggests that enlarged supratentorial ventricles contribute to an increase in ICV. Conversely, supratentorial parameters like LVV showed significant negative correlations with infratentorial parameters like PCFV, CBMV, and BSV, consistent with previous findings [25]. As noted in the introduction, enlarged supratentorial ventricles (hydrocephalus) may be considered a consequence of a small posterior cranial fossa. Considering the benefits of prenatal closure for MMC fetuses- such as higher resolution rates for hindbrain herniation [37] or cerebellar ectopia [8] and a 50% lower shunt placement rate [37–39]-it is evident that there is a close relationship between the supratentorial and infratentorial malformations caused by MMC.

In MMC cases, thoracic-level lesions were associated with smaller CBMV compared to lumbar/sacral lesions [24], and lower MMC levels correlated with reduced incidence of CM-II [25]. Increased ventricular size on fetal MRI has been linked to larger spinal defects [9]. In our study, CM-II infants exhibited a higher prevalence of thoracic lesions, lower sacral lesions, and greater MMC lesion vertebrae counts than non-CM-II infants. Our correlation analysis showed that higher MMC lesion vertebrae counts were associated with smaller PCFV and larger LVV. We inferred that more severe spinal conditions lead to more severe intracranial malformations.

Our volumetric comparison between non-CM-II and CM-II cases corroborated previous findings, indicating a smaller PCFV in CM-II [24, 25]. Additionally, our study demonstrated a larger ratio of CBMV + BSV to PCFV in CM-II compared to non-CM-II infants. This suggests reduced CSF space in the posterior fossa associated with CM-II-related posterior fossa constriction, as indicated by an absence of visible CSF signal [40]. Despite our expectation that PCFV would most effectively distinguish non-CM-II from CM-II, LVV exhibited superior performance in ROC analysis. The well-established association between CM-II and hydrocephalus [3, 13, 22, 25, 40] is further supported by our findings, offering new insights into this relationship.

The rate of CM-II-related operations in MMC cases has been reported to range from 9.2% [15] to 19.0% [12], whereas in our study, it was 7.7%. Although we found no volumetric differences between CM-II cases that underwent an operation and those that did not, our ROC analysis highlighted that BSV was most effective in distinguishing between these groups. An autopsy showed severe bulbar derangement [13], and infants and children younger than two years old often exhibit cranial nerve and brainstem signs [14], such as dysphagia [6]. These results align our ROC findings with established evidence. However, it’s important to note that the sample size of CM-II cases requiring surgery was small (*n* = 4).

Our study has several limitations. First, CT scans used for volumetric calculation should ideally have been obtained before closure surgery to exclude or minimize the effects of the surgery. However, in five cases, CT scans were performed after surgery. Additionally, five different CT protocols were used, and we were unable to ensure consistency in the slice width of CT images for volume calculation. Second, since the parts of the cerebellar displacement through the foramen magnum were excluded from the volumetric calculations, CBMV may have been underestimated. Given that previous studies have used MRI for volume calculation [25], MRI provides sufficient spatial resolution to detect herniated cerebellum. However, in this study, obtaining a head MRI at 0 months for all cases was challenging. Third, to establish precise diagnostic criteria, we focused solely on the presence of cerebellar displacement. As a result, MMC infants with CM-II-specific imaging findings other than cerebellar displacement were classified as non-CM-II, which could lead to a lower CM-II rate. Finally, it is a single-center, retrospective analysis without standardized criteria for CM-II-related surgeries. The decision for surgery was based on the clinical judgment of neurosurgeons at our institution.

## Conclusions

This study offered a comprehensive assessment of both supratentorial and infratentorial volumes in MMC infants, revealing significant volumetric differences between non-CM-II and CM-II groups. A notable negative correlation was observed between supratentorial and infratentorial structure volumes. LVV was identified as the most effective parameter for distinguishing between non-CM-II and CM-II infants. These findings emphasize the importance of evaluating CM-II through a combined assessment of both supratentorial and infratentorial malformations.

## Electronic supplementary material

Below is the link to the electronic supplementary material.

Supplementary Material 1

## Data Availability

The data used in this study are available from the corresponding authors upon reasonable request and after additional ethics approval.

## Abbreviations

AUC: Area under the curve
BSV: Brainstem volume
CBMV: Cerebellum volume
CM-II: Chiari malformation type II
COV: Cut-off values
c.p: Corrected p-values
CSF: Cerebrospinal fluid
CT: Computed tomography
DICOM: Digital Imaging and Communication in Medicine
ICV: Intracranial volume
LVV: Lateral ventricles volume
mL: milliliter
MMC: Myelomeningocele
MRI: Magnetic resonance imaging
PCFV: Posterior cranial fossa volume
ROC: Receiver operating characteristic
T1WI: T1-weighted image
T2WI: T2-weighted image
VPS: Ventriculoperitoneal shunt
