# Supplementary material for: Comprehensive assessment of supratentorial and infratentorial volumes in infants with myelomeningocele with and without Chiari malformation type II
Source: Neuroradiology. 2024 Dec 4;67(3):755–65. doi: 10.1007/s00234-024-03514-9 (PMC12003494; doi:10.1007/s00234-024-03514-9)

Supplemental Table 1 CT scan protocols employed in this study.

CT scans assessed in this study were acquired using five different protocols, including four helical scans and one volume scan. As no records of CT scans prior to 2011 were available, data from 13 scans are missing. The volume scan protocol was employed for children with difficulty controlling body movements. Helical scans are classified into four types based on tube current (either 200 mA or 150 mA) and the use of auto exposure control (AEC). Radiation doses are reported as the mean volume computed tomography dose index ($CTDI_{vol}$) with standard deviation. All CT scans were acquired with a 0.5mm slice width, and the images were reconstructed with the following values: 1 mm (n = 5), 3 mm (n = 3), 5 mm (n = 42), and 6 mm (n = 2).

**Scanning type**	**Helical #1 (n=15)**	**Helical #2 (n=9)**	**Helical #3 (n=6)**	**Helical #4 (n=1)**	**Volume (n=8)**
**Tube voltage [kV]**	120	120	120	120	120
**Tube current [mA]**	200	150	200	150	80~200
**Rotation time [s]**	0.6	0.6	0.6	0.6	1
**Pitch factor [mm]**	0.641	0.641	0.641	0.641	1
**Collimation [mm]**	0.5 × 64	0.5 × 64	0.5 × 64	0.5 × 64	0.5 × 320
**Slice spacing [mm]**	0.5	0.5	0.5	0.5	0.5
**AEC**	on	on	off	off	−
**$CTDI_{vol}$ [mGy]**	20.60 ± 5.85	20.31 ± 3.33	38.60 ± 0.00	28.90 ± 0.00	18.06 ± 2.76

Supplemental Fig.1 Cerebellar displacement through the foramen magnum.

A sagittal midline view of the T1-weighted MRI obtained 18 days after birth (15 days after closure surgery) is shown in (a), with cerebellar displacement indicated by a red arrow. An axial view of CT scans obtained two days after birth (one day before the closure surgery) is shown in (b), with the slice level indicated by a blue line in panel (a). In panel (b), the herniated cerebellum is suspected to be presented; however, differentiating between the herniated cerebellum and the cervical spinal cord is challenging.

CT, computed tomography; MRI, magnetic resonance imaging.

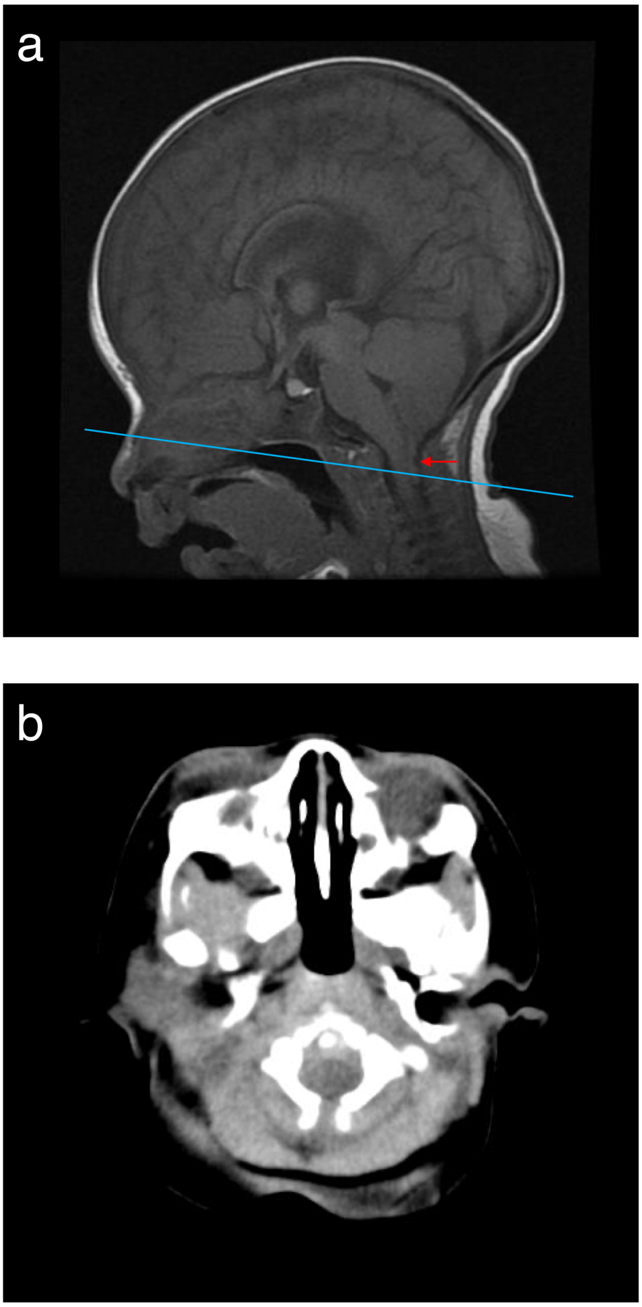

Supplemental Fig.2 Flowchart of diagnosis.

We used the first head CT scans obtained after birth for volumetric calculations. Based on these CT scans, radiologists at our hospital determined the diagnosis of non-CM-II and CM-II by assessing the presence of cerebellar displacement. Of the 52 CT scans, 47 were performed before closure surgery, with 43 being sufficient for diagnosis. However, for nine cases, a head MRI was necessary for a definitive diagnosis. All MRIs were performed after closure surgery.

Diagnoses were made by either one or two radiologists, and the mean years of experience in pediatric radiology are presented with standard deviations. When a single radiologist made the diagnosis, they had over 20 years of experience. In cases involving two radiologists, the younger radiologist had approximately 10 years less experience than the senior radiologist. Diagnoses made by younger radiologists were reviewed and confirmed by the senior radiologists.

CM-II, Chiari malformation type II; CT, computed tomography; MRI, magnetic resonance imaging.

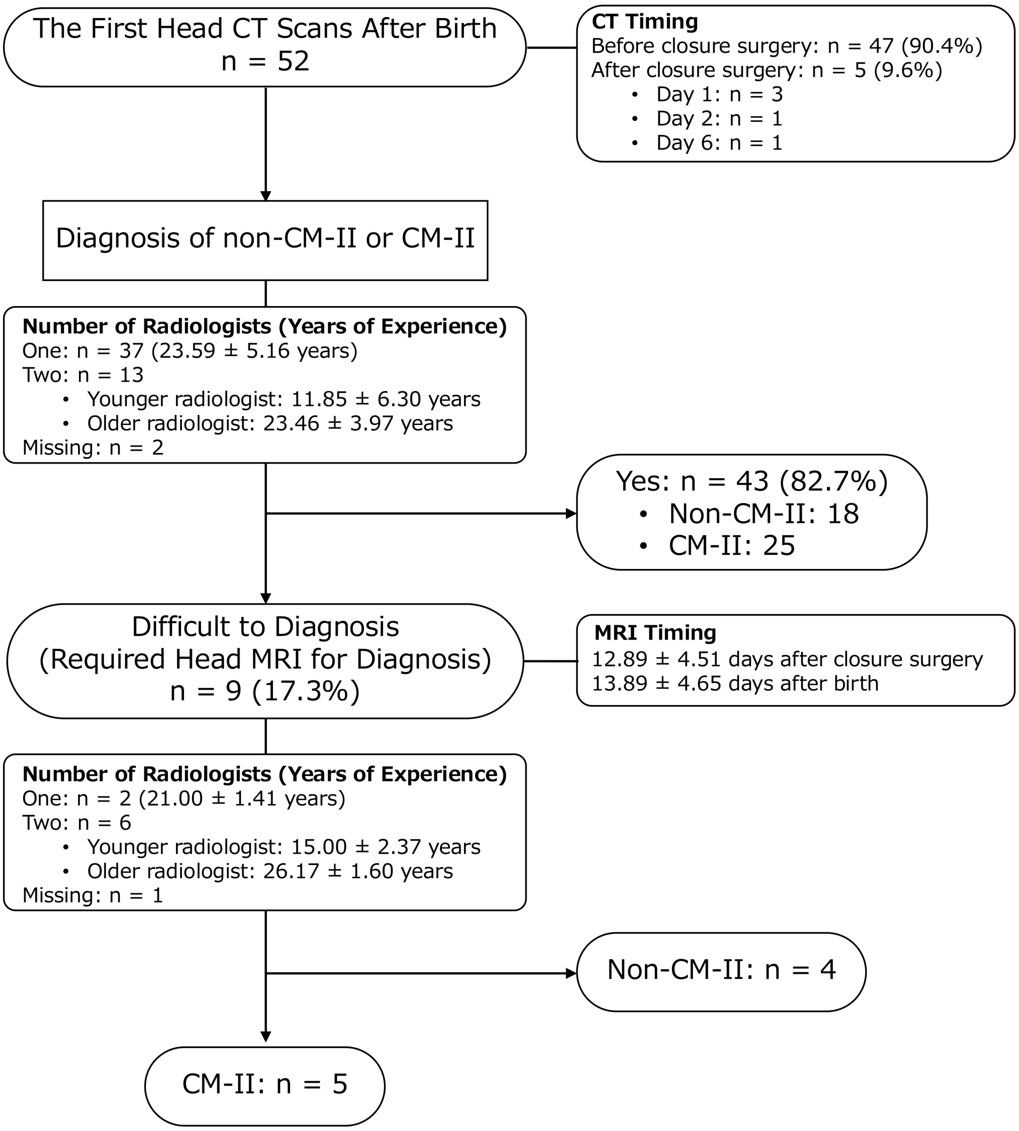

Supplemental Fig.3 Patients enrollment in this study.

A total of 52 infants were analyzed, although data on gestational week and cesarean section were missing in some cases. Of these, 30 infants were diagnosed with CM-II, and 22 were classified as non-CM-II, according to our diagnostic criteria. MMC infants without cerebellar displacement through the foramen magnum were classified as non-CM-II, while those with cerebellar displacement were classified as CM-II. Further details can be found in the "Patients and study setting" subsection of the Material and Methods section. Four patients required FMD with C1 laminectomy.

CM-II, Chiari malformation type II; FMD, foramen magnum decompression; MMC, meningomyelocele.

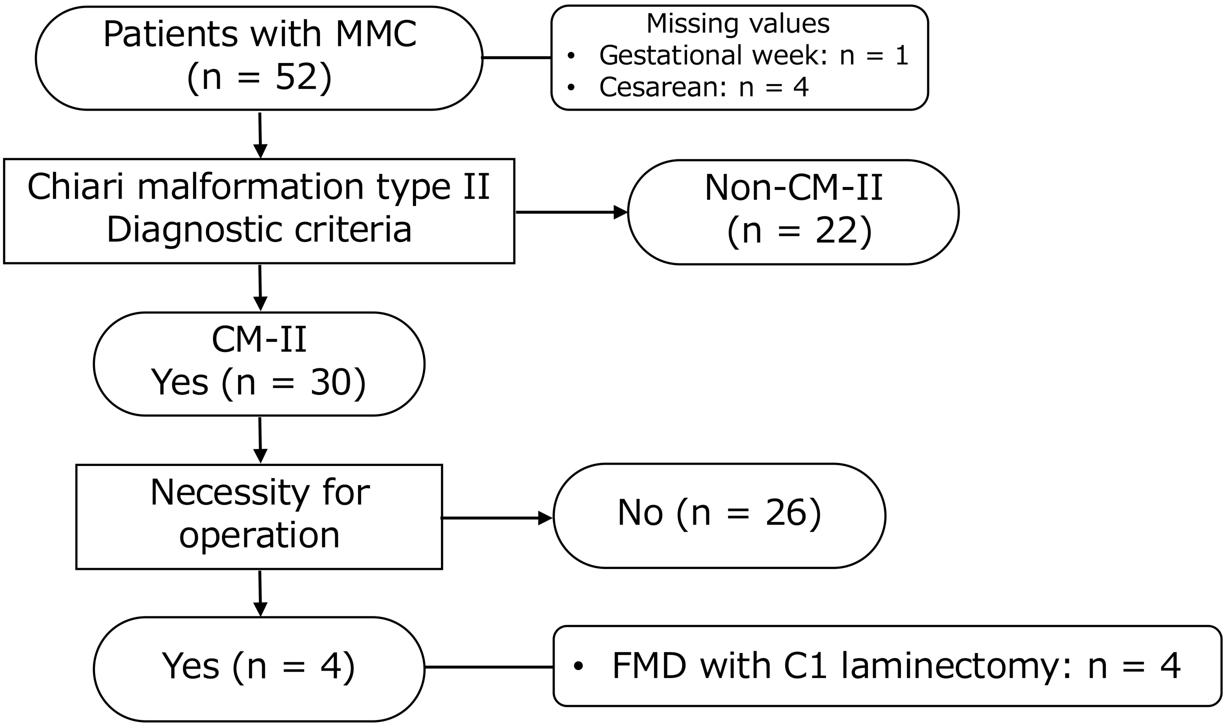

Supplemental Fig.4 Flowchart for fetal diagnosis.

Fetal diagnoses were made in 37 cases, with fetal MRIs performed in 32 of these cases. One or two radiologists made diagnoses, and the mean years of experience in pediatric radiology are presented with standard deviations. Diagnoses made by younger radiologists were reviewed and supported by senior radiologists. Among the 24 cases diagnosed as CM-II based on fetal MRIs, 21 were confirmed as CM-II after birth. Of the 8 cases diagnosed as non-CM-II in fetal MRIs, 5 were confirmed as non-CM-II after birth. Therefore, in 26 out of 32 cases (81.3%), fetal diagnoses were consistent with postnatal diagnoses.

CM-II, Chiari malformation type II.

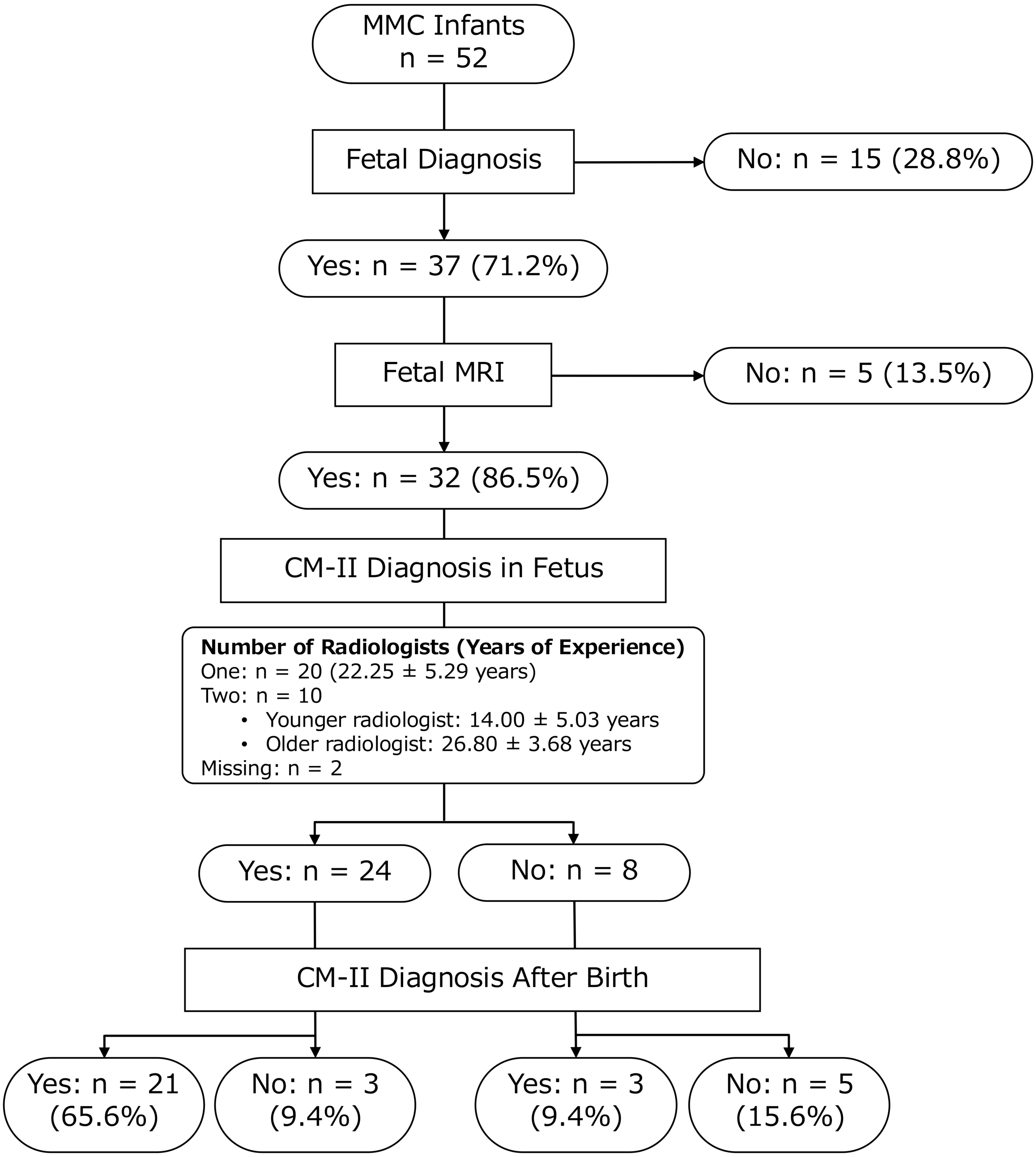

Supplemental Fig.5 Distributions in three-dimensional space.

Each individual's data is plotted in three-dimensional space: LVV, PCFV, and MMC lesion vertebrae count. Data from non-CM-II and CM-II are colored blue and red, respectively.

CM-II, Chiari malformation type II; LVV, lateral ventricles volume; MMC, myelomeningocele; PCFV, posterior cranial fossa volume.

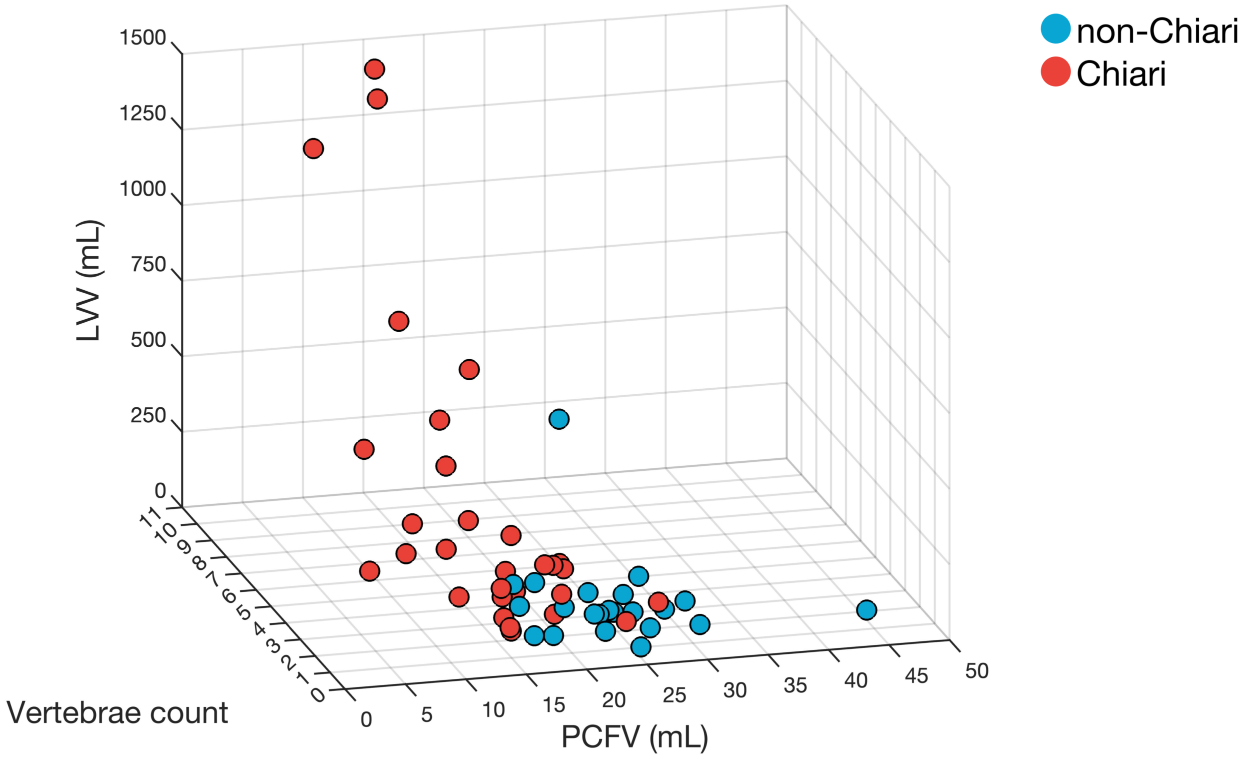

Supplement: Supplementary file 1 — Supplementary Material 1 [file 234_2024_3514_MOESM1_ESM.pdf]
